# Age and Glaucoma-Related Characteristics in Retinal Nerve Fiber Layer and Choroid: Localized Morphometrics and Visualization Using Functional Shapes Registration

**DOI:** 10.3389/fnins.2017.00381

**Published:** 2017-07-12

**Authors:** Sieun Lee, Morgan L. Heisler, Karteek Popuri, Nicolas Charon, Benjamin Charlier, Alain Trouvé, Paul J. Mackenzie, Marinko V. Sarunic, Mirza Faisal Beg

**Affiliations:** ^1^Faculty of Applied Sciences, School of Engineering Science, Simon Fraser University Burnaby, BC, Canada; ^2^Center for Imaging Sciences, Johns Hopkins University Baltimore, MD, United States; ^3^Institut Montpelliérain Alexander Grothendieck, CNRS, Université de Montpellier Montpellier, France; ^4^CMLA, ENS Cachan, Centre National de la Recherche Scientifique, Université Paris-Saclay Cachan, France; ^5^Department of Ophthalmology and Visual Sciences, Faculty of Medicine, University of British Columbia Vancouver, BC, Canada

**Keywords:** optical coherence tomography, computational anatomy, Bayesian estimation, retina, glaucoma, aging

## Abstract

Optical coherence tomography provides high-resolution 3D imaging of the posterior segment of the eye. However, quantitative morphological analysis, particularly relevant in retinal degenerative diseases such as glaucoma, has been confined to simple sectorization and averaging with limited spatial sensitivity for detection of clinical markers. In this paper, we present point-wise analysis and visualization of the retinal nerve fiber layer and choroid from cross-sectional data using functional shapes (fshape) registration. The fshape framework matches two retinas, or generates a mean of multiple retinas, by jointly optimizing the surface geometry and functional signals mapped on the surface. We generated group-wise mean retinal nerve fiber layer and choroidal surfaces with the respective layer thickness mapping and showed the difference by age (normal, younger vs. older) and by disease (age-matched older, normal vs. glaucomatous) in the two layers, along with a more conventional sector-based analysis for comparison. The fshape results visualized the detailed spatial patterns of the differences between the age-matched normal and glaucomatous retinal nerve fiber layers, with the glaucomatous layers most significantly thinner in the inferior region close to Bruch's membrane opening. Between the young and older normal cases, choroid was shown to be significantly thinner in the older subjects across all regions, but particularly in the nasal and inferior regions. The results demonstrate a comprehensive and detailed analysis with visualization of morphometric patterns by multiple factors.

## Introduction

Volumetric optical coherence tomography (OCT) has emerged as a preferred diagnostic tool in ophthalmology for noninvasive, *in-vivo*, micrometer-resolution imaging of the eye. Recent progress in OCT imaging has allowed the acquisition of highly detailed 3D images from which morphometric measurements can be derived. Optic nerve head measurements and the thickness of the peri-papillary retinal layers have been used clinically to detect and monitor glaucoma progression (Leung, 2014). While these measurements are useful individually, a lack of clear spatial references for biomarkers limits the spatial and anatomical correspondence across multiple images. For example, in the common sectoral layer thickness analysis of OCT scans, the peri-papillary area is split into quadrants (superior, inferior, nasal, and temporal) which are then further subdivided into circumferential areas. This analysis relies on averaging over the local sectors to reduce noise, and to mitigate potential inconsistency in sectoral placement across individuals. Such a sectoral averaging approach is limited by a minimum size of sectors to achieve comparisons in the same vicinity in the different individuals. Hence, the sectorization approach reduces the spatial sensitivity of the measurements due to averaging over larger areas and could potentially impact clinical assessment. This motivates the need to develop tools that can generate measurements on a point-to-point basis, eliminating the need to average data in local regions.

Most previous studies involving registration of OCT images have averaged multiple serially acquired images for noise reduction and motion correction (Jørgensen et al., 2007; Young et al., 2011), or utilized rigid alignment of time-course images (Niemeijer et al., 2009). Relatively little attention has been given to registration of cross-sectional OCT data. Chen et al. (2014) performed intensity-based non-rigid registration of macular OCT scans by a combination of rigid alignment of foveae, and A-scan-wise affine and non-rigid registration using radial basis functions for refined alignment of the retinal layers (Chen et al., 2014). A more recent work (Anthony et al., 2016) by the same group applies this registration technique to perform voxel based morphometry in macular OCT of healthy controls and multiple sclerosis patients. Our group's approach has focused on retinal surface-based registrations and atlas generation. In (Gibson et al., 2010), 3D optic cup surfaces were registered to a single template surface, first by rigid and nonrigid intensity-based volumetric registration, followed by spherical mapping and spherical demons registration of the surfaces. This work was further expanded upon in Lee et al. (2015) which represented the retinal surfaces utilizing the framework of mathematical currents. Two surfaces were brought into close proximity by minimizing a functional of reproducing kernel Hilbert space norm-based energy and a dissimilarity term, then registered by spherical demons to establish homology. More recently, we introduced the functional shape (fshape), framework (Charlier et al., 2015; Lee et al., 2017). In this framework, the retinal surface (shape or geometry) and any value mapped on the surface, for example, retinal layer thickness (function or signal), are considered together as a single mathematical object called *fshape*. One fshape can be matched to another or the mean of multiple fshapes can be computed by joint optimization of the energies associated to varifold-based dissimilarity measures of geometry and function. For group analyses, the algorithm can generate population atlases and establish homology across the database, facilitating comparison of morphometric measurements in localized regions. Moreover, fshape mean computation or matching can be performed with flexible constructions of fshapes that can include multiple geometric shapes and function parameters; this allows the building of specific sets of geometry and function features to compare across multiple groups.

In this paper, we demonstrate the use of this algorithm for investigating the effect of age and glaucoma on retinal nerve fiber layer (RNFL) and choroid. Loss of RNFL in the optic nerve head (ONH) region is a well-known hallmark of glaucoma that leads to irreversible vision loss (Medeiros et al., 2005). Currently, the RNFL thickness profile along a circular scan centered at the ONH and the sectoral average thickness maps are used in clinics to assess the disease progression. Studies have shown regional patterns in glaucomatous RNFL thinning, with most significant changes in the inferior peripapillary region (Leung et al., 2010; Mwanza et al., 2011). Aging has been also associated with RNFL loss (Budenz et al., 2007; Parikh et al., 2007). The effect of glaucoma in the choroid has been more debated, with some studies reporting glaucoma-related thickness changes (Song et al., 2016; Li et al., 2017), and others reporting no changes (Ehrlich et al., 2011; Maul et al., 2011). Recent works on OCT angiography (Lee E. J. et al., 2016; Mammo et al., 2016) suggest vascular impairment in glaucoma, and this motivates simultaneous, localized analysis of the two layers to investigate possible connection in the structural changes due to glaucoma.

In this work, we aim to (i) examine the spatial RNFL thickness patterns by age and by presence of glaucoma by comparing the reference group of older healthy eyes with younger healthy eyes and with age-matched glaucoma eyes, and (ii) study whether there is spatial relationship between age-related changes and glaucoma-related changes.

## Materials and methods

### Participants and image acquisition

Thirty-eight eyes from five young healthy participants, five older healthy participants, and twelve older glaucoma patients were included in the study. The participant demographics are listed in Table 1. Before being included in the study, each participant was subject to optic nerve head OCT imaging, dilated stereoscopic examination of the optic nerve, stereo disc photography, intraocular pressure (IOP) measurement, and reproducible Humphrey perimetry at the Eye Care Center at Vancouver General Hospital. Eyes with retinal disease other than primary open-angle glaucoma, uveitis, IOP lower than 10 mmHg or greater than 20 mmHg, or optic neuropathy from causes other than glaucoma were excluded. The mean glaucoma duration at the time of imaging was 3.69 ± 3.80 years. A custom 1,060-nm swept-source OCT system by the Biomedical Optics Research Group at SFU was used for imaging. Each image consisted of 400 B-scans, with 400 A-scans per B-scan and 1,024 pixels per A-scan. The axial voxel resolution was 2.7 μm, the axial coherence length was ~6 μm, and the lateral pixel resolution ranged from 11.9 to 14.5 μm depending on the eye's axial length. The A-scan rate of 100 kHz resulted in ~1.6 s of acquisition time per volume.

**Table 1:** Participant demographics.

**Group**	**N (Subjects)**	**N (Eyes)**	**Female/male**	**Mean age**
Young healthy	5	10	3/2	29.8 ± 3.6
Older healthy	5	10	2/3	57.0 ± 4.4
Older glaucoma	12	18	6/6	61.7 ± 7.9

### Preprocessing, segmentation, and layer thickness measurement

**Figure 1:**
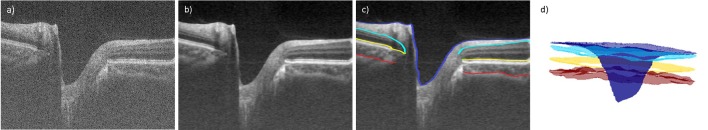
Example OCT B-scan **(a)** without processing, **(b)** after BV smoothing, and **(c)** with the segmentation of inner limiting membrane (purple), posterior boundary of retinal nerve fiber layer (cyan), Bruch's membrane (yellow), Bruch's membrane opening (green), and choroid-sclera boundary (red). 3D surface generation of the retinal layer boundaries is shown in **(d)**.

Images with artifacts, such as large lateral motion artifact or the ONH not being at the center of the field of view were excluded from this analysis. The image underwent axial motion correction by B-scan cross-correlation and 3D bounded-variation smoothing for reducing the effect of speckles and enhancing the visibility of anatomical structures, with no additional normalization. An example of OCT B-scan before and after processing is shown in Figures 1a,b. Retinal nerve fiber layer (RNFL) and choroid were segmented automatically by delineating inner limiting membrane (ILM), RNFL-ganglion cell layer boundary, Bruch's membrane (BM), and the choroid-sclera boundary using a 3D graph-cut based algorithm (Li et al., 2006; Lee et al., 2013a,b). All automated segmentation results were checked by a trained rater, and incorrect segmentation was manually corrected using Amira (version 5, FEI). Bruch's membrane opening (BMO) was manually segmented on 80 radial slices extracted from the image volume. An example of the segmentation is shown in Figures 1c,d. A best-fit 3D BMO ellipse was computed using principal component analysis and least square fitting. The segmented RNFL and choroid were cropped at 0.25 mm from the BMO ellipse to account for the ambiguity in the retinal layer boundary close to the optic cup (Lee et al., 2014). Recent studies reported on inconsistencies resulting from using the conventional optic cup as a reference due to its ambiguous definition based on 2D projection fundus images, and showed the BMO was an viable alternative reference as a robust anatomical structure defined in 3D space (Chauhan and Burgoyne, 2013; Gardiner et al., 2014). The layer thickness was measured at each point as the closest 3D Euclidean distance between the posterior and anterior surfaces of the layer. Prior to the fshape registration step, all corresponding surfaces were rigidly aligned by matching the BMO ellipse centroid.

### Fshape registration

The framework of functional shapes (or fshapes) provides a quantitative measure of inter-subject variability in the RNFL and choroid. In this section, we briefly summarize the algorithm which is detailed in Lee et al. (2017). Let the *i*th subject's RNFL or choroid thickness be represented by (*X*^*i*^, *f*^*i*^), where *X* is the layer surface (geometry) and *f* is the surface-indexed function (thickness here) mapped on *X*. Let a template exemplar for this database be denoted by (*X*_∗_, *f*_∗_), consisting of a template surface geometry *X*_∗_ and an associated surface-indexed signal (thickness here) mapping *f*_∗_. Given (*X*_∗_, *f*_∗_) as the template fshape, and (*X*^*i*^, *f*^*i*^) as the *i*th target fshape to be registered to the template, the fshape framework will estimate a smooth deformation ϕ^*i*^ of the template geometry *X*_∗_ and a residual ζ^*i*^ to be added to the template function *f*_∗_, such that after transformation of the template fshape with the mapping ϕ^*i*^ to the *i*th target fshape, the geometry of the transformed template matches the geometry of the *i*th target $X^{i}\approx \phi^{i}\left(X_{*}\right)$ and the transformed template function plus residual matches the function of the *i*th target i.e., $f^{i}\approx \left(f_{*}+\zeta^{i}\right)◦{\phi^{i}}^{-1}$. Hence, for each of the target fshapes (*X*^*i*^, *f*^*i*^) for all *i* = 1..*N* a pair (ϕ^*i*^, ζ^*i*^) consisting of a smooth deformation and a function residual are estimated such that:

(1) $$\begin{array}{l} \left(X^{i},f^{i}\right)\approx \left(\phi^{i},\zeta^{i}\right)\;\cdot \;\left(X_{*},f_{*}\right)=\left(\phi^{i}\left(X_{*}\right),\left(f_{*}+\zeta^{i}\right)◦{\left(\phi^{i}\right)}^{-1}\right)\\ \;=\left({\tilde{X}}^{i},{\tilde{f}}^{i}\right) \end{array}$$

**Figure 2:**
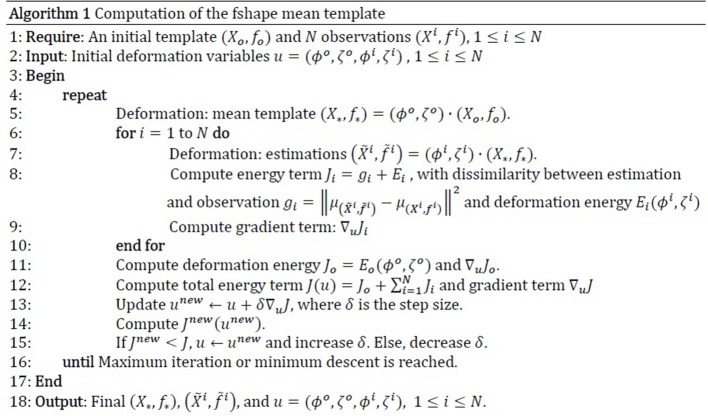
Computation of fshape mean template.

The function residual added to the template function estimated by fshape matching effectively becomes the representative of the target function in the geometry of the template. By fixing a template geometry and function, a group of target fshapes can be brought into the coordinates of the template such that the residuals representing different target function values are now indexed on the same template geometry. The choice of the template is an important consideration and the mean of the observations in the database is a standard choice. A database fshape mean is hence estimated, to be used as a template, by an adaptive gradient descent algorithm that jointly optimizes a total fshape dissimilarity measure between each target fshape (*X*^*i*^, *f*^*i*^) and the transformed template $\left({\tilde{X}}^{i},{\tilde{f}}^{i}\right)$ for all *i* taken together. The process is summarized in Figure 2. A similar mean template generation procedure was repeated for the choroid. This maps all the target observations' function values (layer thickness) into a common coordinate system of the mean template where statistical analysis can be applied on the residuals (ζ^i^) to compute the point-wise variability of the function values at each point on the template geometry across the database.

### Sectorization

**Figure 3:**
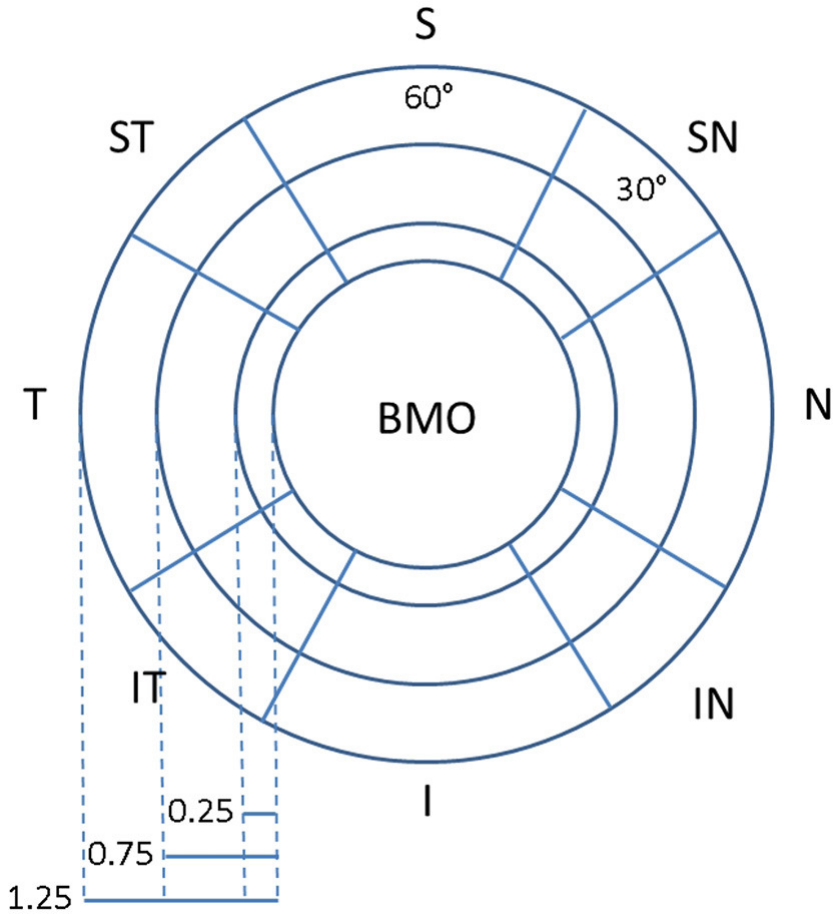
Sectorization of the layer surface with reference to the Bruch's Membrane Opening (BMO) as reference.

In order to compare the fshape analysis with more conventional “intrinsic” analysis where an individual-specific coordinate system is placed on each geometry separately such as using sectoral analysis, the peri-papillary retinal layers were divided into regional sectors (Lee et al., 2014) as shown in Figure 3. Unlike standard sectors defined by fixed distances from the center of the optic disk on the enface projection image that do not take into account the individually varying sizes of Bruch's membrane opening (BMO), the sectors in this study were defined in 3D in each eye by the distance from the BMO, which is a more reliable anatomical landmark than the optic disk (Lee et al., 2014). This normalizes the sectors for different retinal tilts and BMO/optic disks sizes. The sectors were first delineated by elliptical annuli at constant distances (0.25, 0.75, 1.25 mm) from the BMO ellipse. These were further divided by superior, nasal, inferior, and temporal sectors, and additionally into superior-nasal (SN), inferior-nasal (IN), inferior-temporal (IT), and superior-temporal (ST) sectors. The first four sectors are 60° wide and the latter are 30° wide. For each sector, the thickness measurements for all points in the sector for that individual eye are taken and averaged to create one scalar number representing the average sectoral thickness value.

### Statistical analysis

#### Group analysis

Two analyses were conducted (1) the fshape analysis of computing the mean fshape geometry and function and the residual function for each subject indexed on the common mean template, enabling point-wise comparison across the subjects in the database and (2) sectoral averages within each subject that enable comparison across subjects by the sectors defined over the subject's layer geometry.

The database consisted of members from three groups: young normal (Group A), older normal (Group B), and older glaucomatous (Group C) individuals. These groups enable analyses of two main questions (1) the effect of age on RNFL and choroid layer thicknesses in healthy young and older individuals (Group A and B comparison) and, (2) the effect of glaucoma between age-matched individuals (Group B and Group C comparison). These two questions were analyzed by point-wise (fshape) and sector-wise group mean thickness difference maps and two-sample *t*-test maps.

#### Regression analysis

The effect of age and glaucoma on RNFL and choroidal thicknesses was additionally examined by point-wise and sector-wise linear regression to quantify trend, on Group A and B for the effect of age, and Group B and C for the effect of glaucoma. Each layer thickness (RNFL or choroid) values from Group A and B were fitted to *layer thickness* = a^*^Age + *b* to estimate the rate of change (mm/year) in the cross-sectional healthy subjects. To assess change as a function of visual field mean deviation (VFMD), a measure of glaucomatous loss measured in decibels (dB), the layer thickness (RNFL or choroid) values from Group B and C were fitted to *layer thickness* = a^*^VFMD + *b* to estimate the rate of change per VFMD (mm/dB).

### Point-wise visualization of RNFL and choroid thickness pattern

To visualize the measurements across the database highlighting the variability and trends, the fshape measures for each subject on the common template were normalized by using a z-score computed by subtracting the mean of a reference group and dividing by the standard deviation of the measures in the reference group. The z-score was calculated point-wise as ${\text{z}}_{\text{k}}=\left({\text{x}}_{\text{k}}-{\overset{®}{\text{x}}}_{\text{k}}^{\text{h}}\right)/\sigma_{\text{i}}^{\text{h}}$, where x_k_ is the thickness at k^th^ point for, ${\overset{®}{\text{x}}}_{\text{k}}^{\text{h}}$ is the mean thickness of the reference group at k^th^ point, and $\sigma_{k}^{h}$ is the standard deviation of the reference group at k^th^ point. The reference groups were chosen to be Group A for age comparison and Group B for glaucoma comparison such that the average measures of the young normal (Group A) or older healthy (Group B) subjects are removed to visualize the residual effects of age and glaucoma, respectively. To present a compact visualization, measurement of each subject was unraveled into a column format by subdividing the mean template surface into sectors, and subdividing each sector into smaller sub-sectors, and arranging the z-score values by their sectors in a column format consistent across the database while preserving spatial adjacencies.

## Results

**Figure 4:**
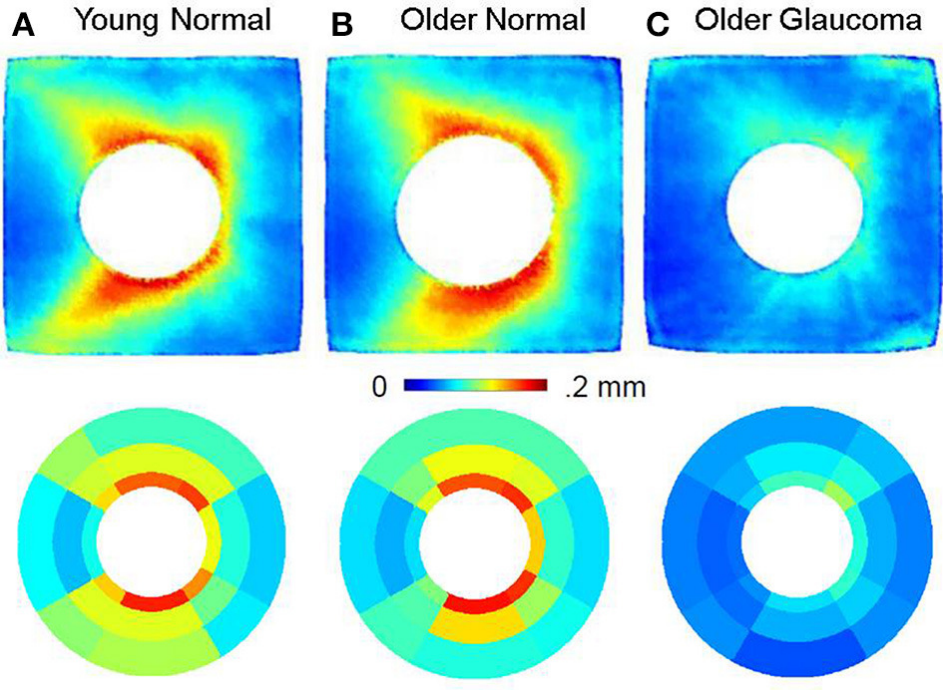
Retinal nerve fiber layer (RNFL) thickness using fshapes point-wise registration **(top row)** and sectorization **(bottom row)** for Group A (Young Normal), Group B (Older Normal), and group C (Older Glaucoma). The RNFL thickness in Group B is similar to Group A, indicating that RNFL is relatively better preserved with age, whereas in Group C, this layer undergoes marked thinning. All images are in the right-eye orientation.

This section will present the results of point-wise (using fshape) and sectoral analysis of RNFL and choroid thickness across the three cohorts chosen for this study. All figures are right-eye oriented, with the left side temporal and the right side nasal. Group averages of RNFL thickness are visualized in Figure 4. The top row shows the point-wise average computed using fshapes and the bottom row shows the results using sectoral averaging. The point-wise fshape mean RNFL templates display detailed salient features from multiple, cross-sectional eyes in each group showing the characteristic hourglass pattern of healthy RNFL in both Group A (young normal) and Group B (older normal) whereas Group C (older glaucoma) show marked glaucomatous thinning. There is good correspondence between the fshape mean templates constructed from point-wise registration and sectoral average maps taken from unregistered RNFL thickness averages in each sector in each individual.

**Figure 5:**
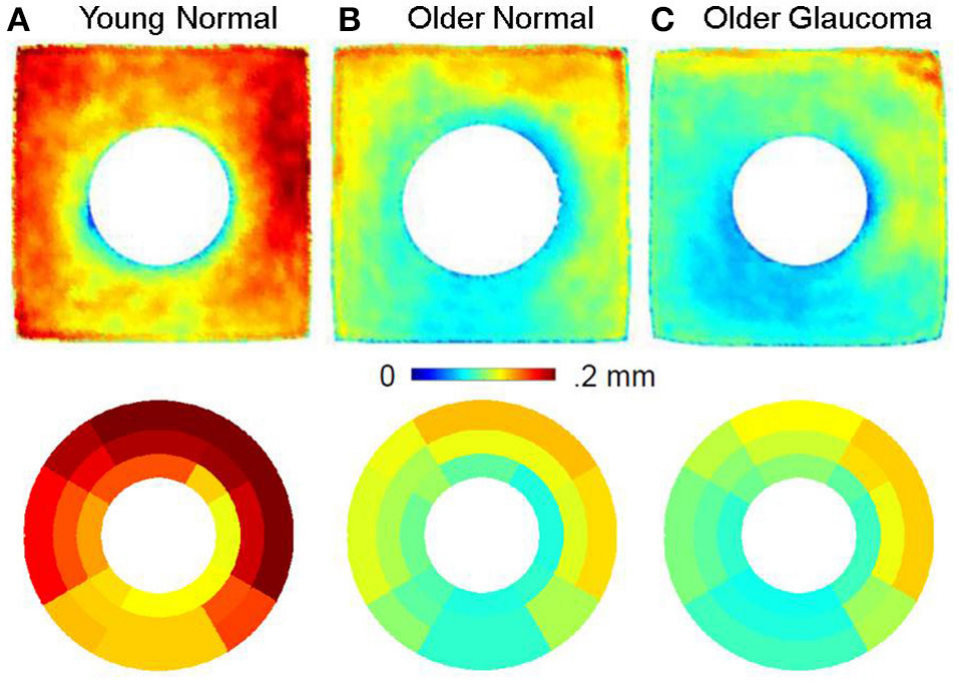
Choroid thickness using fshapes **(top row)** and sectorization **(bottom row)** for Group A (Young Normal), Group B (Older Normal) and Group C (Older Glaucoma). The choroid in the older normal subjects is markedly thinner than the choroid in the younger normal subjects revealing a thinning process that seems to be based on chronological age. All images are in the right-eye orientation.

The group averages of choroidal thickness by fshape (top row) and sectoral averaging (bottom row) is shown in Figure 5. All three groups display thicker choroid in the nasal and superior regions and thinner choroid in the inferior region. The choroid is visibly thinner with age as seen in Group B compared to Group A. As with RNFL thickness, there is overall correspondence between the fshape mean templates and sectoral average maps.

**Figure 6:**
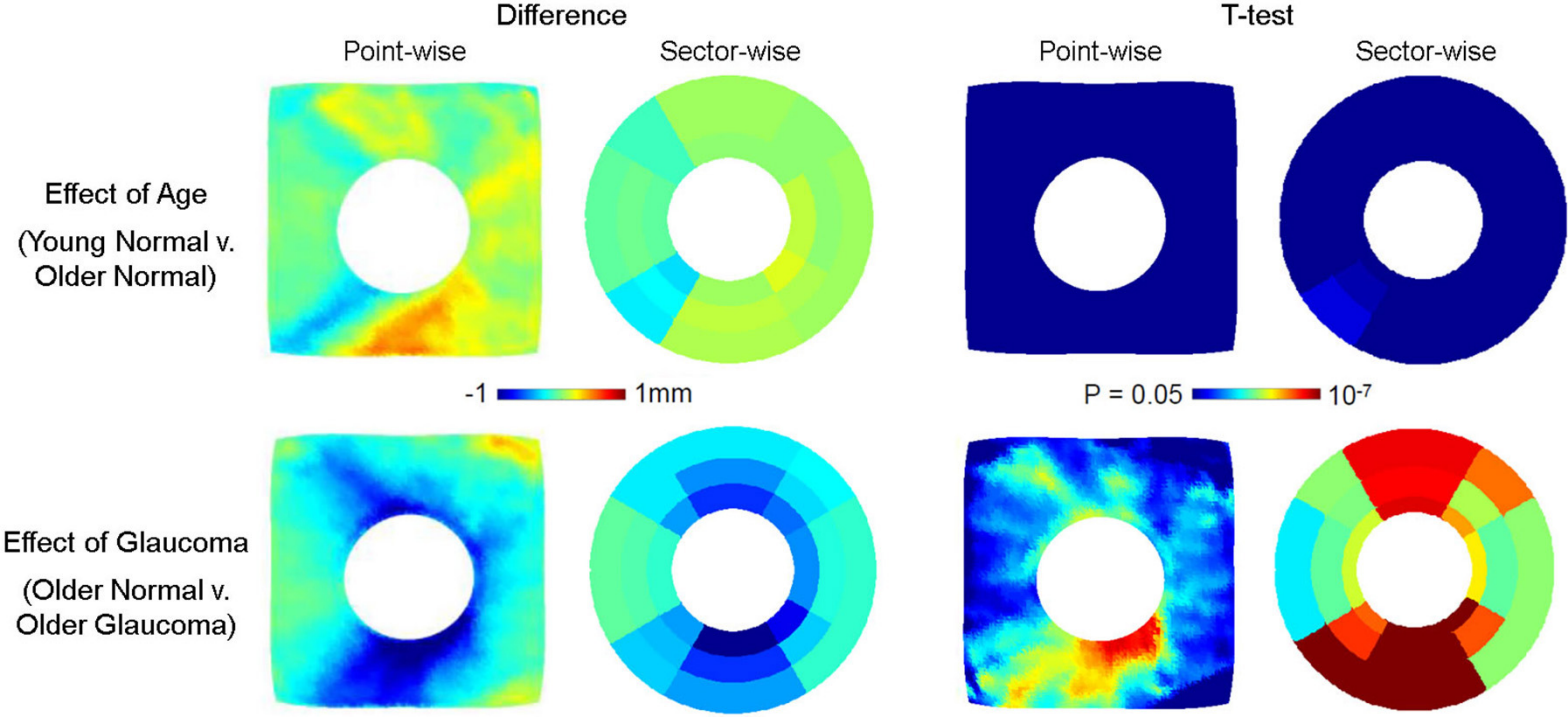
Effect of age (top row) and glaucoma (bottom row) on RNFL thickness. The point- and sector-wise subtraction of mean RNFL thickness between older normal (Group B) and young normal (Group A) is shown in the top left panel, and the *p*-values from point- and sector-wise *t*-test is shown in the top right panel. The point- and sector-wise subtraction of RNFL mean between older glaucoma (Group C) and older normal (Group B) is shown in the bottom left panel, and the *p*-values from point- and sector-wise *t*-test is shown in the bottom right panel. The RNFL layer is found not to change significantly with age, whereas it changes significantly with glaucoma. The fshapes point-wise comparison shows the pattern of change in greater detail than the sectorization, revealing the localized pattern of glaucomatous RNFL thinning. All images are in the right-eye orientation.

The effect of age and glaucoma in RNFL thickness is shown by difference of group averages in Figure 6. The top row shows the effect of age in RNFL thickness in the healthy subjects comparing the mean RNFL thickness in young normal vis-a-vis the older normal individuals. The bottom row shows the effect of glaucoma in RNFL thickness by comparing age-matched older subjects with and without glaucoma. The left panel shows the point- and sector-wise difference of RNFL thickness from Group B by Group A (top, young vs. older) and from Group C by Group B (bottom, healthy vs. glaucoma) at each corresponding points / sectors. The right panel shows point and sector-wise *t*-test results indicating where the group difference is significant. The RNFL thickness is found to not change significantly over age across individuals (top row), whereas the difference due to glaucoma is apparent (bottom row). The loss of RNFL thickness is observed to be higher in regions where normal RNFL thickness is higher and suggests some regional correspondence between the degree of glaucomatous RNFL loss and the original RNFL thickness. The *t*-test significance map between the healthy and glaucoma subjects shows the highest statistical significance in the inferior region.

**Figure 7:**
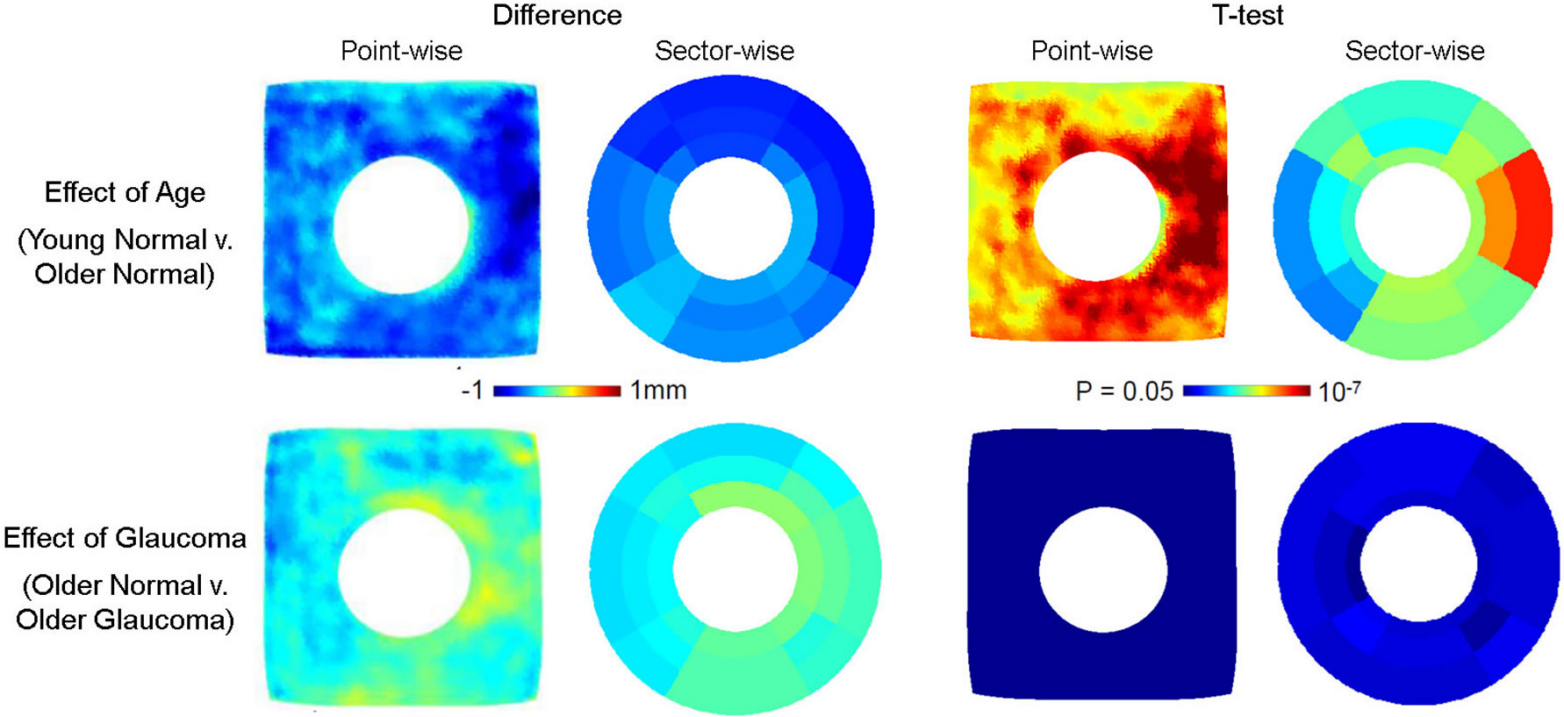
Effect of age (top row) and glaucoma (bottom row) on choroidal thickness. The point- and sector-wise subtraction of mean choroidal thickness across older normal (Group B) and young normal (Group A) is shown in the top left panel, and the *p*-values from point- and sector-wise *t*-test is shown in the top right panel. The point-and sector-wise subtraction of mean choroidal thickness between older glaucoma (Group C) and older normal (Group B) is shown in the bottom left panel, and the *p*-values from point- and sector-wise *t*-test is shown in the bottom right panel. The choroid is found to thin significantly with age, whereas changes are relatively less with glaucoma. The fshapes point-wise comparison shows the pattern of change in greater detail, revealing the localized pattern of age-related choroidal thinning. All images are in the right-eye orientation.

Similar group difference visualization for choroid is shown in Figure 7. Compared to RNFL, choroidal thickness is more different by age (top row, Group B − Group A) than by glaucoma (Group C − Group B). Choroidal thickness of Group B is consistently lower than that of Group A across all regions, but in particular in the nasal and superior regions. The locations of statistical significance, as shown in the *t*-test map, of the age-related group difference is found particularly in the nasal region. The choroidal thickness difference by glaucoma was not as strong as that due to age and although the choroidal thickness in Group B was overall larger than that of group C, the point- and sector-wise *t*-test show limited statistical significance.

**Figure 8:**
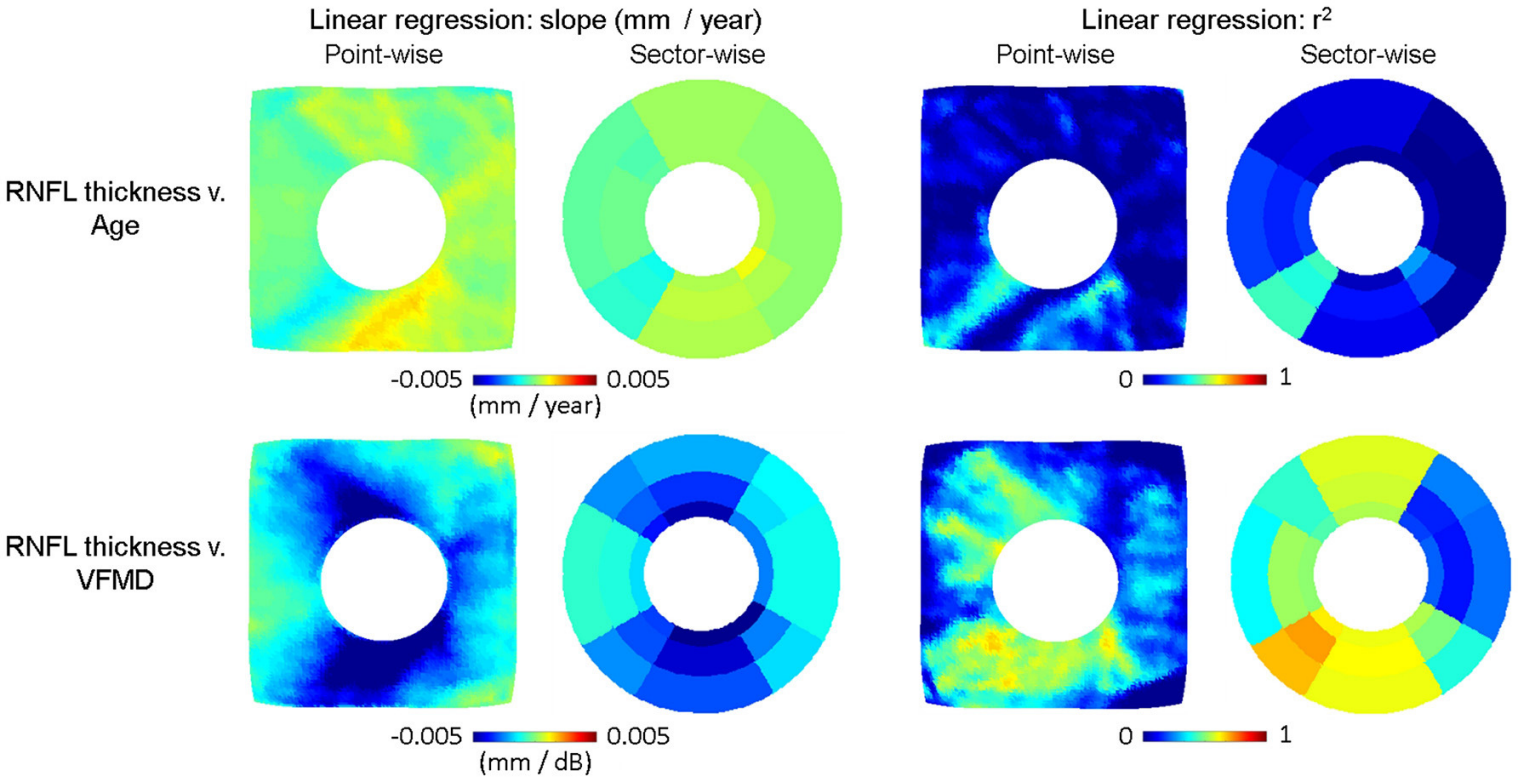
Linear regression plots of RNFL thickness v. age (top row) and v. visual field mean deviation (VFMD). The slope in RNFL thickness = a^*^Age + b is plotted point- and sector-wise in the top left panel, along with the goodness of fit (*r*^2^) in the top right panel. The slope of RNFL thickness = a^*^VFMD + b is plotted point- and sector-wise in the bottom left panel, along with the goodness of fit (*r*^2^) on the bottom right panel. As with the group difference, RNFL thickness is negatively correlated with VFMD. The point-wise maps reveal that the rate of change has distinct spatial pattern with greater thinning along the regions where RNFL is generally thicker in healthy subjects. All images are in the right-eye orientation.

The relationship of RNFL thickness to aging and severity of glaucoma was examined by point- and sector-wise linear regression in Figure 8. The estimated slope or the rate of change associated with aging (mm/year) in the cross-sectional healthy subjects was plotted in the top row along with the goodness of fit by *r*^2^. The estimated slope or the rate of change associated with visual field loss (mm/dB) in the cross-sectional age-matched subjects was plotted in the bottom row along with the goodness of fit by *r*^2^. Aging did not show consistent, significant trend with RNFL thickness, whereas increasing glaucoma severity (more negative VFMD) was correlated to decreasing RNFL thickness. Similar spatial patterns are observed as seen in the group difference maps shown in Figure 6. In the most severely affected regions of superior-temporal and inferior-temporal regions, RNFL thickness change per MD unit decrease exceeded 5 μm.

**Figure 9:**
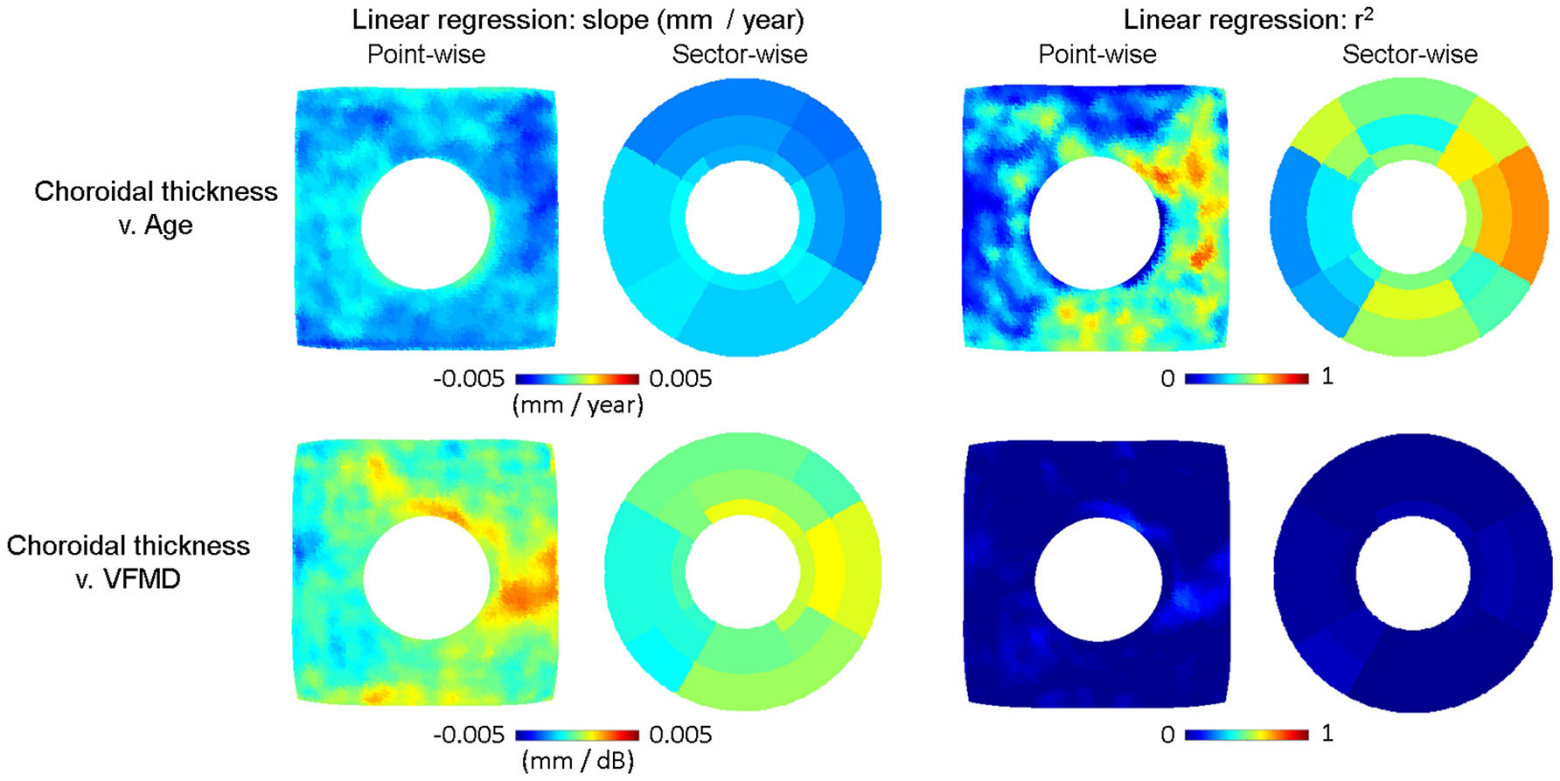
Linear regression plots of choroidal thickness v. age (top row) and v. visual field mean deviation (VMFD). The slope in Choroidal thickness = a^*^Age + b is plotted point- and sector-wise in the top left panel, along with the goodness of fit (*r*^2^) in the top right panel. The slope of Choroidal thickness = a^*^VFMD + b is plotted point- and sector-wise in the bottom left panel, along with the goodness of fit (*r*^2^) on the bottom right panel. As with the group difference, choroidal thickness is negatively correlated with age. The point-wise maps show the steepest change in the nasal and inferior regions. All images are in the right-eye orientation.

The relationship of choroidal thickness to aging and severity of glaucoma was also examined by point- and sector-wise linear regression in Figure 9. The estimated slope or the rate of change associated with aging (mm/year) in the cross-sectional healthy subjects was plotted in the top row along with the goodness of fit by *r*^2^. The estimated slope or the rate of change associated with visual field loss (mm/dB) in the cross-sectional age-matched subjects was plotted in the bottom row along with the goodness of fit by *r*^2^. Again, compared to the RNFL thickness, change in the choroidal thickness was associated more with aging than glaucoma. Aging was correlated to globally decreasing choroidal thickness, most significantly in the nasal and inferior regions. In the most severely affected regions, the average choroidal thickness change per year was ~3–4 μm.

The point-wise nature of fshape metrics can be utilized by simultaneous visualization of all data points from multiple subjects. Figure 9 displays the RNFL fshape thickness in the order of VFMD, which measures the glaucomatous functional loss, for the age-matched normal and glaucomatous eyes of Group B and C. In the top panel, each column represents an eye's point-wise RNFL fshape thickness, which approximates the true RNFL thickness as the sum of the RNFL fshape mean template thickness and the residual (t_i_ ≈ f_∗_ + ζ_i_) at each point. Horizontally, the eyes are ordered by VFMD, plotted below the thickness plot in grayscale. Vertically, the points are ordered by sectors from Nasal (N) and counter-clockwise to Inferior Nasal (IN). Within each sector, the points are ordered by the distance from BMO center, from the closest to the farthest. This visualization allows one to compare all eyes at each spatial location. There is observed an overall group-wise difference between Healthy older subjects, Early Glaucoma, and Moderate to Severe Glaucoma subjects. Comparing vertically from the top to the bottom, the healthy eyes show the thickness pattern that follows the mean template for Group B in Figure 4 where the superior and inferior regions are the thickest, and within each region, RNFL is thicker toward BMO and thins farther from BMO.

**Figure 10:**
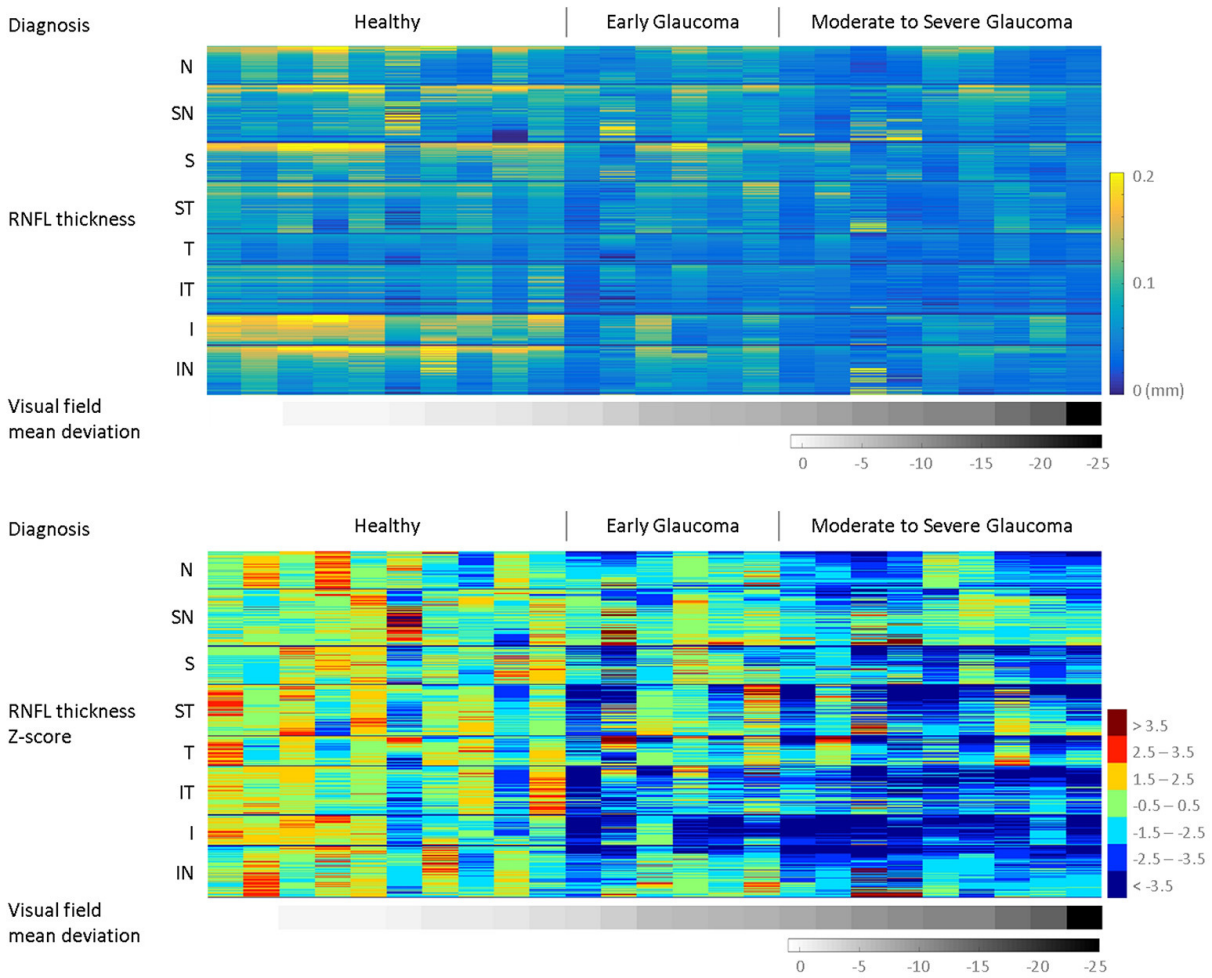
Multi-subjects RNFL thickness by visual field mean deviation (VFMD). By mapping to the common template space, all subjects have estimated thickness values (t_i_ ≈ f_∗_ + ζ_i_) at the same corresponding spatial points, visualized in the top panel. Vertically, the points are ordered by regions, from Nasal (N) to Inferior Nasal (IN). Within each region, the points are ordered by their distance from BMO center, from the closest to the farthest. Horizontally, the eyes are ordered by VFMD, plotted in grayscale below the thickness plot. In the bottom panel, the same data is visualized z-scores, which normalizes the values by the mean and standard deviation of the reference group (Group A, young healthy) and highlights the trend across regions and increasing VFMD magnitude.

The bottom panel of Figure 10 visualizes the f-shape thickness in the top panel using z-scores normalized by the group mean and standard deviation of the RNFL thickness of the young healthy group (Group A) at each point, with increasing magnitude indicating greater deviation from the reference group. The differences between the groups are more apparent in z-score plot, with clear regional characteristics. Regionally, inferior RNFL, and to a lesser degree, inferior-temporal RNFL, are consistently thinner in all glaucoma eyes. In other sectors, the magnitude of z-score is greater for the moderate to severe glaucoma group than early glaucoma. The plot also shows glaucomatous RNFL thickness change is greater nearer BMO by the vertical gradations within individual regions in the glaucomatous eyes.

**Figure 11:**
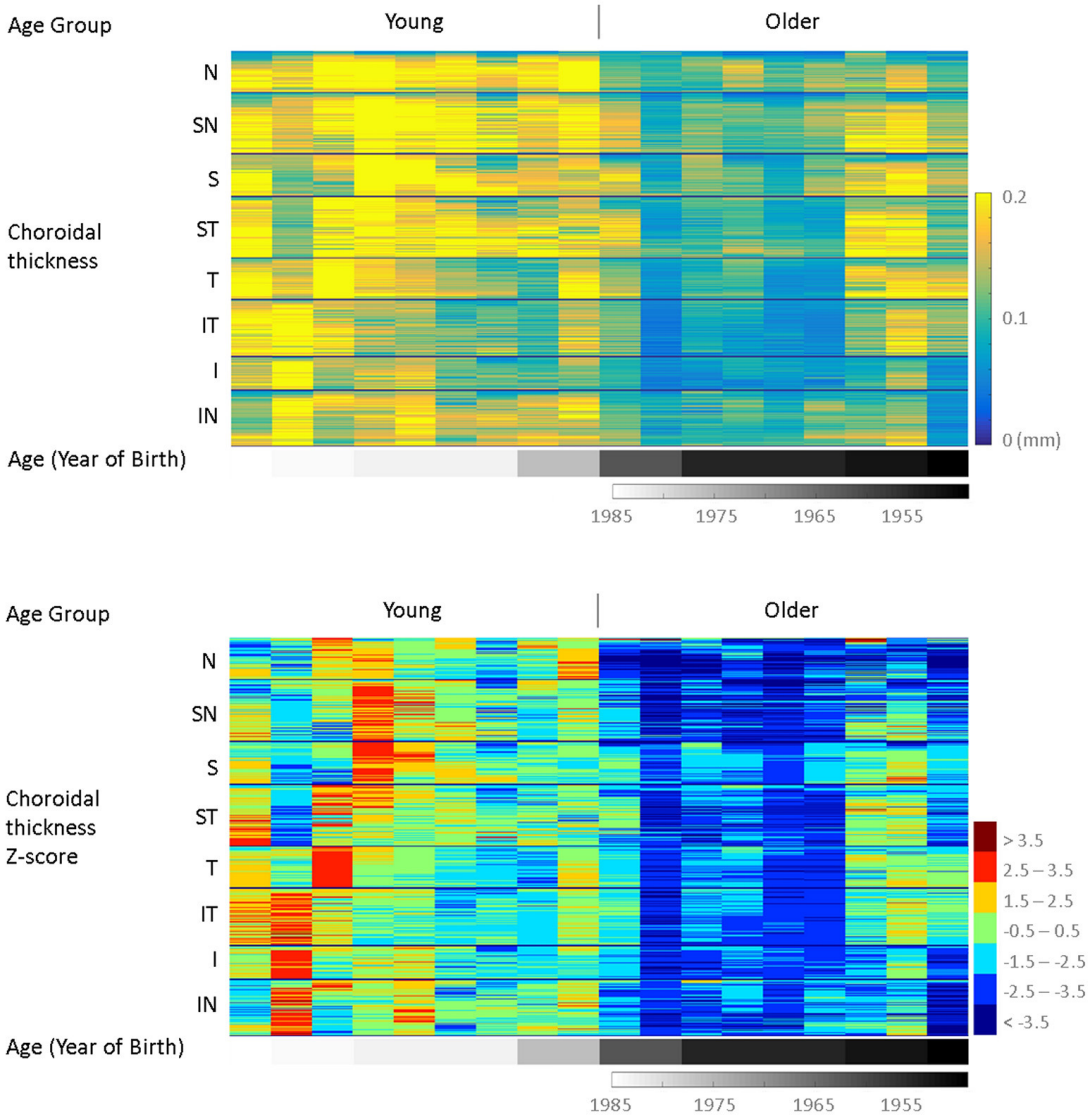
Multi-subjects choroidal thickness by age. By mapping to the common template space, all subjects have estimated thickness values (t_i_ ≈ f_∗_ + ζ_i_) at the same corresponding spatial points, visualized in the top panel. Vertically, the points are ordered by regions, from Nasal (N) to Inferior Nasal (IN). Within each region, the points are ordered by their distance from BMO center, from the closest to the farthest. Horizontally, the eyes are ordered by age, plotted in grayscale below the thickness plot. In the bottom panel, the same data is visualized in z-scores, which normalizes the values by the mean and standard deviation of the reference group (Group A, young healthy), and highlights the trend across regions and increasing age.

Figure 11 displays the choroidal fshape thickness in the order of age for the normal eyes to show the thinning of the choroid observed with age. In the top panel, each column represents an eye's point-wise choroidal fshape thickness, which approximates the true choroidal thickness at each point. Horizontally, the eyes are ordered by age, plotted below the thickness plot in grayscale. As in Figure 10, the points are vertically ordered by sectors from Nasal (N) and counter-clockwise to Inferior Nasal (IN), and within each sector, the points are ordered by the distance from BMO center, from the closest to the farthest. In the bottom panel, the same data is visualized in z-scores normalized by the group mean and standard deviation of the choroidal thickness of the young healthy group. As shown in Figures 5, 7, there is a marked difference between the young healthy and older healthy eyes, and the choroid appears generally thicker in the superior half than the inferior half. Within the young eyes, the z-score is generally lower for the older eyes. As seen in Figure 7, the nasal region of the older eyes shows the highest magnitudes of z-score, suggesting the age-related choroidal thinning may be the most significant in the region.

## Discussion

The 3D OCT images reveal the structure of the ocular posterior segment in great detail so as to enable visualization and quantification of the retinal layer morphometry. Individual measurements of layer thicknesses can be pooled into population-wide assessments of normative layer thicknesses and any changes that may occur as a function of age and disease. These allow insights into whether age and disease have a stereotypical pattern of influence in the retina, with common shape features and localizations, as well as variability across individuals and deviation of a particular subject from a reference population. In this paper, we presented the effect of age and glaucoma on retina nerve fiber layer (RNFL) and choroid using our novel f-shapes approach, which enables a point-wise assessment of retinal morphometrics across individuals via a registration approach. The fshape registration estimates a residual function that is added to the template thickness such that the template geometry after transformation matches the subject geometry, and the template thickness plus the residual function after transformation matches the subject thickness. This maps an individual's layer thicknesses onto the common coordinate system of the template geometry via the specific residual function estimated for that individual. At each location on the template surface, subsequent statistical analysis across the database can reveal trends and features that are common across individuals as a function of age and disease. A more conventional approach utilizes sectorization of the layer surface by calculating the average of all thickness measurements within each sector for each individual eye. Assuming that the sectors are defined with consistent anatomical and spatial correspondence across individuals, within-sector average provides a single scalar summary measure for a given sector that can be statistically analyzed for cross-sectional data taken from the same sector across the individual eyes. However, such approach is limited in spatial sensitivity due to averaging of values in a region. We examined the effect of age and glaucoma in RNFL and choroidal thickness in both of these approaches with four quantitative visualizations: (i) group rages (Figures 4, 5), (ii) group-wise difference and *t*-test maps (Figures 6, 7), (iii) linear regression with age and visual field mean deviation (VFMD) as predictors (Figures 8, 9), and (iv) multi-subject fshape thickness and z-scores plots (Figures 10, 11). The computation time including automated segmentation and mean template generation was ~40 min on a high-performance GPU cluster.

With age, RNFL showed relatively little difference between the young and older healthy subjects, with regression estimating no strong relationship between age and RNFL thickness. In previous studies using OCT measurements, RNFL thickness has been negatively associated with age (Budenz et al., 2007; Parikh et al., 2007; Bendschneider et al., 2010; Sarunic et al., 2010; More et al., 2011). In this study, the mean age of the young healthy subjects was thirty, and that of the older healthy subjects was fifty-seven. The age difference between the two groups may be too small for any marked difference, especially with the small sample size in the study. Older age was, however, associated with markedly thinner choroid, and the point-wise registration showed the difference was more significant in the nasal and inferior regions. With the recent advancement in OCT devices enabling the posterior boundary of the choroid to be imaged, age-related choroid thinning has been reported by multiple groups (Manjunath et al., 2010; Maul et al., 2011; Barteselli et al., 2012). Our result suggests overall thinning of peripapillary choroid with age, but with regional differences. That the older healthy subjects had comparable RNFL but thinner choroid compared to the young healthy subjects may indicate the age-related choroidal thinning does not directly and concurrently impact RNFL thickness. Recent work using speckle-variance OCT (SV-OCT) (Mammo et al., 2016) has shown degradation of RNFL microcapillaries in glaucoma, and it has been suggested the glaucomatous tissue loss may be driven by changes to the microvasculature. Although the choroid is a vascular layer, it mainly supplies the outer layers of the retina that are unaffected in glaucoma, and may therefore be separate from the factors that drive the RNFL tissue and capillary loss in glaucoma.

Glaucoma, as expected, was observed to be associated with significant thinning of RNFL. The pattern of loss visualized in the point-wise maps forms the hourglass crescent-shaped ridge, particularly in the inferior arm, highest toward the middle of the ridge, decreasing further away from the ridge center. The same hourglass-like pattern is observed in young normal subjects. The results of our study are consistent with known patterns of RNFL loss in glaucoma, but more importantly, it shows that the glaucomatous RNFL loss occur in a specific, uneven pattern that follows the initial RNFL thickness, suggesting that the time of onset and significance of the RNFL loss may be proportional to the initial RNFL thickness in the region. The loss significance was also higher in the region closer to BMO. The fshape RNFL loss map elucidates the results of previous studies that found the highest diagnostic ability of the RNFL loss in the inferior and temporal-inferior sectors (Sehi et al., 2009; Mwanza et al., 2011). Although the choroidal thickness in the older glaucoma subjects seemed to be somewhat less than in the age-matched healthy subjects, it was still statistically comparable, indicating that glaucoma pathology may not have significant, direct impact on the choroid as it does on the RNFL. The role of choroid in glaucoma has been debated, and it is likely complex and multifaceted. Disturbed autoregulation of the choroid has been suggested as part of the disease pathology (Hayreh, 1969; Ulrich et al., 1996). Multiple studies using OCT images (Ehrlich et al., 2011; Maul et al., 2011; Li et al., 2013) consistently reported no changes in the peripapillary choroidal thickness in primary open angle glaucoma (POAG); however, Li et al. (2017) reported thicker temporal peripapillary choroidal area in POAG patients using enhanced depth imaging OCT, and Song et al. (2016) reported global and all 12 clock-hour peripapillary choroidal thickness thinner in OAG patients using swept-source OCT.

Figures 10, 11 presented a large-data visualization with each subject's point-wise thickness values color mapped in a single column, and multiple subjects' data columns displayed concurrently, arranged in the order of visual field mean deviation (VFMD, Figure 10) and age (Figure 11). This method allows for presentation of all data points from multiple subjects in a way that highlights the trend and discrepancies in the data. Normalizing the data by the mean and standard deviation of the young, healthy group as the reference removes the baseline in the data and further brings out the differences with respect to controls.

The patterns of change shown in the point-wise and sector-wise approaches were overall consistent. The point-wise registration was able to show localized features in higher resolution compared to the sectorization, revealing detailed regional patterns and potentially furthering our understanding of the disease mechanism. This approach may be useful for characterizing the focal localized patterns that are often seen in glaucoma, both in RNFL and in other subsurface structures such as lamina cribrosa and peripapillary tissues. In this work, we also examined multiple factors (age, glaucoma) in multiple layers (RNFL, choroid) concurrently for a more complete picture in understanding our data. The results showed glaucomatous thinning of RNFL, and age-related thinning of choroid and how the spatial patterns of the tissue loss in the two layers were localized and distinct. Limitations of this work include relatively small sample size, inclusion of fellow eyes, and light beam angle-related uncertainty in retinal layer thickness measurement, although given the relatively small size of the field of view in the images in this study, this effect is likely limited. Our future work will include expanding the analyses presented here to other retinal layers and to the macular region to build a comprehensive presentation of the retinal morphometrics, and incorporating retinal vessels and capillary density measures from SV-OCT as metrics along with layer thickness.

## Ethics statement

The study followed the tenets of the Declaration of Helsinki, and informed and written consents were obtained from the participants. Ethics review for the study was approved by Simon Fraser University (SFU) and University of British Columbia (UBC).

## Author contributions

Designed research: SL, KP, NC, BC, AT, PJM, MVS, and MFB. Analyzed and interpreted data: SL and MFB. Wrote manuscript: SL, MLH, MVS, and MFB. Reviewed and approved manuscript: KP, NC, BC, AT, PJM, MVS, and MFB.

### Conflict of interest statement

The authors declare that the research was conducted in the absence of any commercial or financial relationships that could be construed as a potential conflict of interest.
